# The effect of endoscopic transoral outlet reduction (TORe) on weight recidivism and insufficient weight loss following one-anastomosis gastric bypass (OAGB). Tore after OAGB for weight recidivism

**DOI:** 10.1007/s00423-026-04021-6

**Published:** 2026-03-23

**Authors:** Fadi Kinaani, Winnie Mathur, Relly Reicher, Mohit Bhandari, Nathaniel Aviv Cohen, Shai Meron Eldar, Adam Abu-Abeid, Danit Dayan, Manoel Galvao Neto, Sigal Fishman, Mati Shnell

**Affiliations:** 1 Bariatric Endoscopy Unit, Department of Gastroenterology, Tel Aviv Sourasky Medical Center, Faculty of Medicine & Health Sciences, Tel Aviv University, Waizman 6, Tel Aviv, Tel Aviv, 6423906 Israel; 2 Mohak Bariatric and Robotic Center, Bariatric Endoscopy department, Indore, India; 3 Department of Surgery, Sri Aurobindo Medical College and PG Institute, Indore, India; 4 Bariatric Center, Department of Surgery Tel Aviv Sourasky Medical Center, Faculty of Medicine & Health Sciences, Tel Aviv University, Tel Aviv, Israel

**Keywords:** OAGB, Weight recidivism, TORe

## Abstract

**Introduction:**

Obesity remains a global health challenge. One-anastomosis gastric bypass (OAGB) has gained popularity for its advantages, including significant weight loss. However, concerns have arisen regarding weight regain and other complications. This study aims to assess the effectiveness and safety of endoscopic transoral outlet reduction (TORe) as a treatment for weight regain after OAGB.

**Methods:**

In this retrospective study, consecutive patients from two bariatric centers with weight regain following OAGB were evaluated. Eligible patients were those with an increase in weight of 10% or a BMI = > 30. Diagnostic gastroscopy determined anastomosis diameter and rule out contraindications for TORe. The procedure involved full thickness suturing over the anastomosis, following circumferential argon plasma coagulation. Patients data were collected, outcomes assessed in accordance with ethical standards.

**Results:**

64 patients were treated over a period of 4 years and were followed up for a minimum of 1 year. Mean age was 45.2 ± 1.4. Percentages of females were 47%. Mean pre-treatment BMI was 35.5 ± 0.5. Average weight regain from post OAGB nadir was19.4% (112.5Kg from 90.7Kg). Average weight loss following TORe was 7% of total body weight (TBW) after 6 months and 8% after 12 months (*p* = 0.03). Additional treatments were needed in 11 (17%) patients. Two patients suffered a perforation, one of which required surgical intervention.

**Conclusion:**

TORe appears moderately effective for the treatment of weight regain post OAGB, with a 97% safety rate. Sustained weight loss was seen during 1 year of follow up. Further investigation is needed to assess long term durability.

## Introduction

Obesity is a major threat to personal health around the world, with incidence and prevalence that continue to rise [1]. Bariatric surgery remains the most effective treatment in appropriate patients. It shows reduction in body weight as well as associated comorbidities and over-all mortality [2]. In recent years, one-anastomosis gastric bypass (OAGB), first described by Rutledge in 1997 as the Mini-gastric bypass, has gained popularity, making it the third most common bariatric procedure worldwide [3]. Its advantages include significant weight loss, shorter procedure time, and low rate of early complications [4]. However, concerns have been raised due to a high rate of nutritional deficiencies, steatorrhea, marginal ulcers, gastro-esophageal reflux disease (GERD), biliary reflux (BR), and like most other procedures, weight recidivism (WR) [5].

The true incidence and prevalence of WR after bariatric surgeries are difficult to measure due to the multitude of definitions in use [6]. The overall long term WR for bariatric surgery is estimated to be 50% [7], with 37% after Roux-en-Y Gastric Bypass (RYGB) [8]. Recently, OAGB was shown to have less WR compared to sleeve gastrectomy (SG) and RYGB in approximately 5.7% and 3.6%, respectively [9], however, it still carries a significant WR rate [9]. Indeed, recent publications reported that 7-34.7% of OAGB revisions are due to insufficient weight loss or weight recidivism [10].

Post-operative factors associated with weight recidivism after RYGB include larger gastro-jejunal anastomosis diameter, longer post-operative follow-up, binge eating, increased food urges and lower physical activity [11]. Traditionally the gastro-jejunal anastomosis is wider in OAGB compared to RYGB to allow for bile clearance from the gastric pouch and the main mechanism for weight loss is attributed to the length of the biliary limb [12]. However, the anastomotic size cannot be entirely excluded as a factor in weight recidivism.

While surgical technique has evolved over the years in order to address the issue of WR [13], a few alternative approaches were shown to be effective in treating WR like pharmacotherapy [12] and bariatric endoscopy, both of which should be offered before repeat surgery [14]. Pharmacotherapy was shown to be effective in treating weight recidivism, with GLP-1 receptor agonists being the prominent option [15]. However, side effects, intolerance, costs and long term adherence of these medications remain an issue [16].

In recent years, the endoscopic armamentarium for the treatment of bariatric surgery complications has expanded and now includes a myriad of devices for various indications [17]. Endoscopic transoral outlet reduction (TORe) has emerged as an effective treatment for weight recidivism after RYGB, as well as for dumping syndrome [18]. In addition, reducing the diameter of the anastomosis by TORe was shown to improve symptomatic biliary reflux after OAGB [19]. The rationale to employ TORe in WR is to restrict and delay gastric emptying by narrowing the gastro-jejunal anastomosis and the distal gastric pouch [20]. However, a correlation between the diameter of the anastomosis and weight changes in OAGB has not been demonstrated [21]. We hypothesized that narrowing the pouch outlet would aid in treating WR after OAGB and maintain sustained weight loss.

## Aims

The primary aim was to assess the effectiveness of TORe for the treatment of WR in patients after OAGB. Secondary aims were to assess the safety and durability of this procedure.

## Materials and methods

Patients with WR after OAGB or insufficient weight loss were referred to the bariatric endoscopy clinics in two tertiary referral centres in Tel Aviv, Israel and Indore, India between 08/2018 and 11/2022. Inclusion criteria were weight recidivism and insufficient weight loss, defined as a ≥ 10% increase from the nadir weight and a BMI above 30 kg/m², respectively. We excluded patients with gastro-gastric fistula, marginal ulcer, and any condition precluding therapeutic endoscopic intervention (such as inability to provide informed consent, or ongoing use of anticoagulants other than aspirin that cannot be discontinued).

**Image 1:**
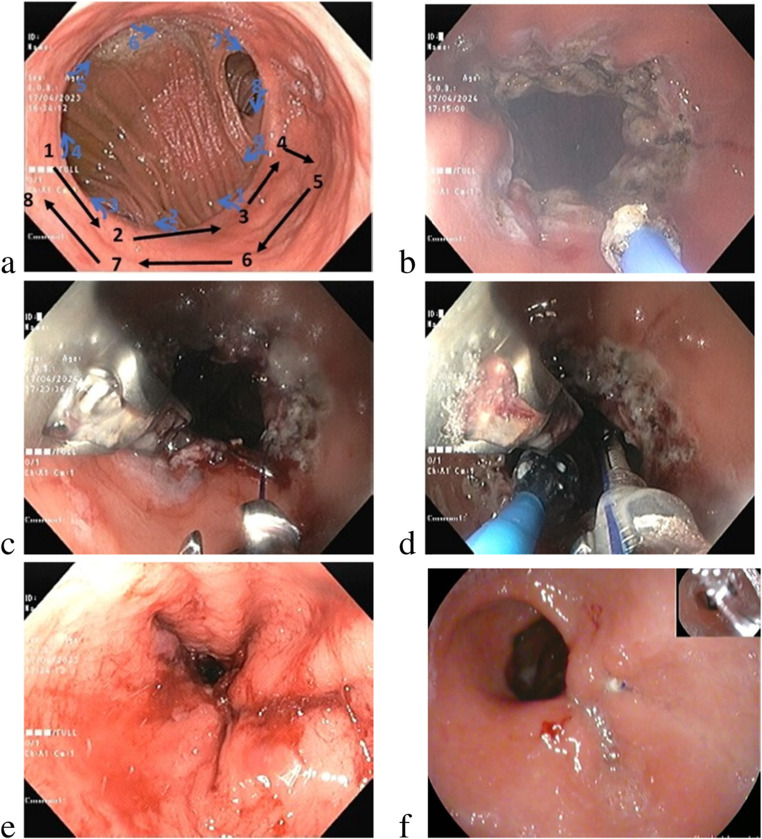
Suture pattern illustration and images of the procedure. **a** Suture pattern – Blue numbers and arrows illustrates the first purse-string suture. Black numbers and arrows illustrates the second "U" suture that imbricates the first suture and re-shapes the distal pouch in a tubular fashion. **b** Circumferential argon plasma coagulation. **c** Purse-string suture. **d** Cinching over an 8 mm balloon (blue catheter). **e** End result after the second U-pattern. **f** Follow up at 6 months

A diagnostic gastroscopy was initially performed to evaluate the diameter of the anastomosis, dimensions of the gastric pouch and to exclude marginal ulcer or gastro-g. astric fistula. TORe was carried out under general anaesthesia, endotracheal intubation, esophageal overtube, and CO2 insufflation in all patients. Before suturing, argon plasma coagulation (APC) was used to cauterize (ERBE 200, 70 W, 0.8 l/min) the circumference of the anastomosis in order to promote scar contraction and further strengthen the narrowing of the anastomosis. TORe was performed using the Apollo Overstich™ (Apollo endo-surgery, Austin, TX) mounted on a double channel gastroscope (Olympus EVIS EXERA II GIF-2TH180 or Fuji ELUXEO El-740). The first suture was placed in a full or semi purse-string pattern across the anastomosis. After placing the suture an endoscopic dilatation balloon (6-7-8 CRE balloon, Boston Scientific) was passed through the anastomosis. The balloon was inflated to 8 mm to allow for calibration and the suture was then cinched over it. A second suture was placed proximally in a “U” pattern, creating an elongated narrow tunnel leading to the anastomosis (Image 1). Additional sutures were used at the discretion of the endoscopist to reduce the anastomosis and\or pouch size. All procedures were done by endoscopists trained in endoscopic suturing. Patients were guided by bariatric dietitians how to gradually transit from liquid diet to solids over a 4-week period. All patients were instructed to continue high-dose PPI for 1 month following the procedure.

### Statement of Ethics

This is a retrospective analysis of prospective cohort, the study protocol was reviewed and approved by Tel-Aviv Sourasky Medical Centre institutional research committee (Affiliation: Tel-Aviv University’s Sackler Faculty of Medicine), approval number TLV-0432-20, and all procedures performed in studies involving human participants were in accordance with the ethical standards of the 1964 Helsinki declaration and its later amendments or comparable ethical standards. Patients were prospectively followed as part of a cohort study authorized by the institutional ethics committee. Researchers were not blinded. Written informed consent was obtained prior to the procedure from all individual participants included in this study. Demographics, clinical and procedural details, and outcomes were gathered and assessed by a physician of the bariatric service, either in person during follow-up visits or by phone interview.

### Statistical Analysis

Values for continuous variables were calculated and expressed as mean ± standard deviation. Categorical variables were presented with their frequencies. Body weight and weight loss were compared at baseline and post-procedure, as described, using paired t test with a two tailed 95% confidence interval and a statistical significance threshold of p < 0.05. Data were statistically analysed using Excel (Microsoft, Redmond, WA, USA) and Prism (version 10.0 for MAC, GraphPad Software Inc., La Jolla, CA, USA).

## Results

Between 8/2018 and 11/2022 TORe was carried out in 64 patients (53% male) with WR or insufficient weight loss after OAGB surgery. At the time of procedure, mean age was 45.1 years (range 19–70) [*CI* 44.85–45.55] and mean BMI was 35.5 kg/m^2^ (range 26.1–48.1) [*CI* 35.38–35.62]. The mean time since OAGB was 3.7 years (range1.5-5) [*CI* 3.66–3.74]. Average WR from the post-operative nadir was 19.4% (range 6.3–42.5%), which corresponded to an average absolute weight gain of 21.8 kg. Mean weight increased from 90.7 kg (at nadir) to a final mean weight of 112.5 kg.

The mean pre-procedure diameter of the gastro-jejunal anastomosis was 29.3 mm (range 25–40 mm) [*CI* 29.18–29.42]. All procedures were technically successful with a reduction of the anastomosis diameter after calibrating with the 8 mm CRE balloon. The average duration of the procedure was 47 min (range 29–66). General patient characteristics and past bariatric interventions are detailed in Table 1.

**Table 1:** Demographic and clinical characteristics of the patient cohort

Total, *n*	64
Gender	
*Female*,* n (%)*	30 (47)
*Male*,* n (%)*	34 (53)
Age (years), mean (±) [CI]	45.2 (1.4) [44.85–45.55]
BMI (kg/m2), mean (±) [CI]	35.5 (0.5) [35.38–35.62]
Years since OAGB, mean (±) [CI]	3.7 (0.1) [3.66–3.74]
Prior intervention	
*LAGB*,* n (%)*	6 (9)
Anastomosis diameter pre-TORE (mm), mean (±) [CI]	29.3 (0.5) [29.18–29.42]

BMI body mass index, OAGB one anastomosis gastric bypass, LAGB laparoscopic adjustable gastric banding, TORE transoral outlet reduction

**Fig. 1:**
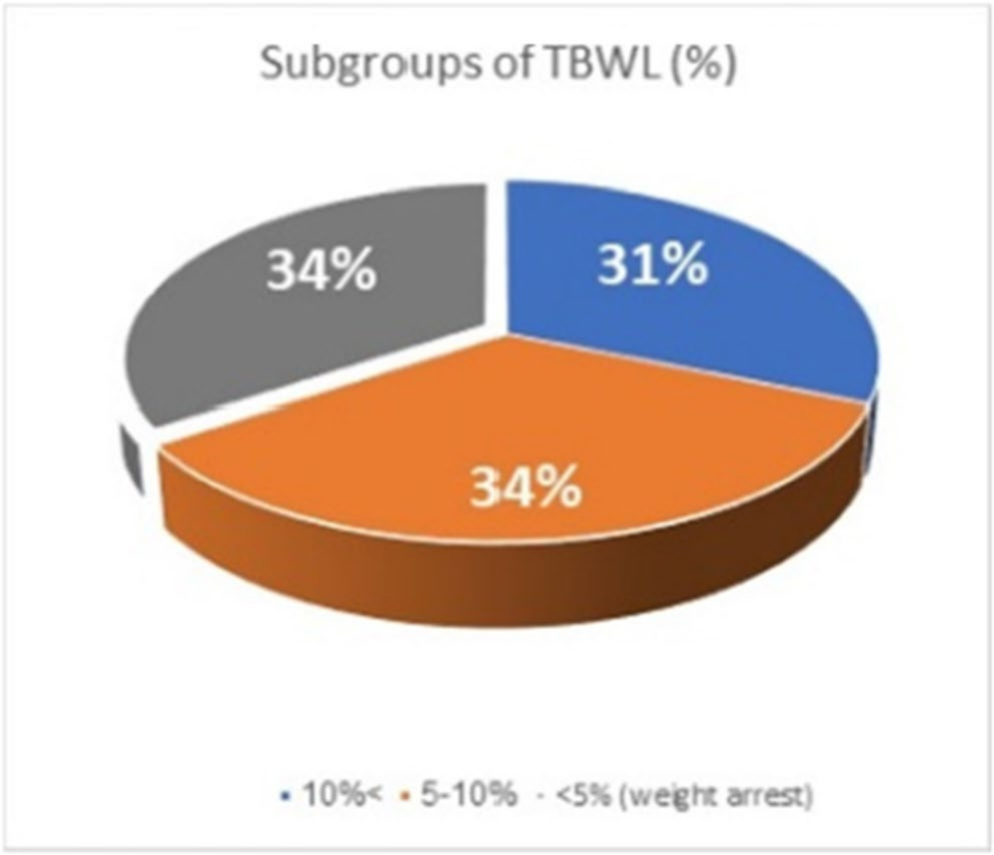
Categorical weight loss groups. TBWL total body weight loss

Patient’s weight was documented 6 and 12 months after TORe and compared to pre-TORe weight. Average total body weight loss after TORe at 6 and 12 months was 6.9 kg or 7% of TBW [*CI* 6.66–7.14] and 8.3 kg or 8% of TBW [*CI* 7.94–8.66], respectively. At one year follow-up 42 (65%) patients lost at least 5%, 22 (34%) patients lost between 5 and 10% and 20 (31%) patients lost more than 10%. Twenty-two patients (34%) lost less than 5% of TBW. This last group of patients were considered “weight arrest” **(**Fig. 1**)**. No patients had weight gain after the procedure..

Notably, weight loss was significantly higher at 1 year follow-up compared to 6 months in terms of both absolute weight and percentage of TBW (8.3 vs. 6.9 *p* = 0.02 and 8% vs. 7%, *p* = 0.03, respectively), Table 2.

**Table 2:** Results at 6 and 12 months

	6 months	12 months	*P*-Value
weight loss (KG), mean (±) [CI]	6.9 (0.4) [6.66–7.14]	8.3 (0.7) [7.94–8.66]	*0.02*
TBWL%, mean (±) [CI]	7 (0.4) [6.76–7.24]	8 (0.6) [7.71–8.29]	*0.03*

TORe transoral outlet reduction, TBWL total body weight loss

Six patients were previously converted from laparoscopic adjustable gastric band (LAGB) to OAGB and, of note, no significant difference was observed after TORe in this group of patients either 6 or 12 months follow-up (8% vs. 7%, *p* = 0.45, and 10% vs. 8%, *p* = 0.68, respectively). Eleven (17%) patients received additional treatment, with glucagon-like peptide 1 (GLP-1) agonists, during the follow up. At 12 months follow-up, the mean total body weight loss (TBWL) was similar between those receiving additional medical therapy and those not (10% vs. 8%, *p* = 0.38). One patient with weight arrest underwent re-TORe for retightening of the dilated anastomosis and had a TBWL of 8%, the same patient was also prescribed a GLP-1 agonist, Table 3.

**Table 3:** Clinical Outcomes and Additional Treatments Following TORe

Parameter	Description	Follow-up (6Mo)	Follow-up (12Mo)	*p*-value	Remarks
Patients previously converted from LAGB to OAGB (*n* = 6)	Total body weight loss (TBWL, %)	8%	10%	6 mo: 0.45 12 mo: 0.68	No significant difference compared with other patients
Patients receiving additional GLP-1 agonist therapy (*n* = 11, 17%)	TBWL (%)	-	10%	0.38	Similar TBWL to patients not receiving additional therapy
Patient undergoing re-TORe (*n* = 1)	TBWL (%)	-	8%		Same patient also received GLP-1 agonist therapy

TORe transoral outlet reduction, TBWL total body weight loss, OAGB one anastomosis gastric bypass, LAGB laparoscopic adjustable gastric banding, GLP-1 Glucagon-Like Peptide-1

Severe adverse events occurred in two cases (3%). Both patients had a perforation of the gastric pouch, presumably from the use of the tissue helix. One perforation was diagnosed during the TORe procedure and treated with endoscopic suturing. A CT scan performed at the end of the procedure ruled out contrast extravasation. The patient was treated with antibiotics and resumed oral diet the next day. No further treatment was needed. The second was diagnosed in the postoperative course. The patient presented with peritonitis, CT scan demonstrated pneumoperitoneum and was treated surgically (Clavien–Dindo classification IIIb). The gastric pouch and the anastomosis were resected, and a gastro-esophageal anastomosis was performed using the remnant stomach. The patient recovered and resumed oral diet.

No other adverse events occurred during the follow-up period, including dysphagia, need for endoscopic dilatation, new onset or worsening GERD, severe pain, bleeding or the need for re-hospitalizaion\re-intervention.

## Discussion

WR may occur in over 50% of patients following bariatric surgery in the long term [22]. The reason for this phenomenon is multifactorial and the size of the GJ anastomosis in RYGB has been shown to be a major factor among others [23]. Scarce data is available regarding surgical treatment of weight recidivism or inadequate weight loss following OAGB. Gastric pouch resizing has been described by Faul et al. [24]. and resulted in BMI reduction of 8 kg/m^2^ at 2 years. Conte et al. described a mean percent excess weight loss of 84% at 2 year follow-up after biliopancreatic limb lengthening following OAGB [25]. Banded bypass, which has also been suggested as an option to reduce the rate of insufficient weight loss [26], may also improve restriction in weight recidivism. However, the morbidity associated with repeat surgical interventions makes endoscopy an attractive therapeutic option in these patients.

TORe was shown to be safe and effective after RYGB with 8–10% weight loss and stabilization of the WR trajectory [27]. In contrast to RYGB, the diameter of the GJ anastomosis after OAGB has not been demonstrated to correlate with weight loss or recidivism. In this study, we have shown that TORe, in patients with WR or insufficient weight loss after OAGB, is moderately effective in achieving weight reduction and trajectory stabilization with comparable magnitude (8% after a year follow up) to reported results of TORe following RYGB. Stabilization of the weight trajectory was achieved in all patients, with 65% of the patients losing at least 5% of their TBW and 35% of the patients losing more than 10%. This is consistent with findings from Musella et al. (2022) [28], who reported a 12-month weight loss of 8.3% following conversion from OAGB to RYGB. Previous smaller series showing the effectiveness of reducing the size of the outlet in alleviating symptoms of biliary reflux [19,21] also demonstrated a positive weight loss effect as secondary outcome. This suggested a possible correlation between the diameter of the anastomosis, the restrictive effect and weight loss. Furthermore, a recent small series of 15 patients demonstrated the effectiveness of TORe after OAGB [29] with an average weight loss of 18.5 ± 2.1% at 1 year, however concurrent use of anti-obesity medications was not specified.

A recent publication demonstrated the advantages of combining endoscopic treatment with anti-obesity medications in TORe [30], reaching an average weight loss of 15.2% ± 7.4% at 1 year. In our series GLP-1 receptor agonists were used in 17% of the patients. These patients experienced improved weight loss, 10% at 1 year compared to 8% in patients not receiving this medication. The difference may be related to the choice of GLP-1 agonist. At the time of the study Tirzepatide and Semaglutide 2.4 mg were still unavailable in India and Israel, so only Liraglutide 3 mg and Semaglutide 1 mg were used. Combining TORe with newer and more potent anti-obesity medications as well as using additional sutures to further reduce the size of the anastomosis may further improve both weight loss and durability, but further research is needed.

One may concern that reducing the anastomosis size in OAGB will aggregate biliary reflux symptoms due to the delayed clearance of bile from the gastric pouch. Importantly, no new onset or worsening of reflux symptoms were reported after the procedure.

In our study 2 perforations occurred (3%), one of them required surgical intervention. Both perforations were related to the use of the tissue helix. Importantly, this adverse event rate is significantly lower than the 14.3% in surgical revisions [31]. However, this does further emphasize the importance of using the helix selectively in revision. While surgical revision with biliopancreatic limb lengthening appears more effective, the endoscopic approach offers a more favorable safety profile [25].

To the best of our knowledge this is the largest series published. Additional strengths of this study are its multicenter design as well as that all procedures were performed by a limited number of experienced bariatric endoscopists. The limitations of this study are its retrospective design, relatively short follow up of up to 1 year, and the non-standardized use of anti-obesity medications.

## Conclusion

In conclusion, TORe appears to be moderately effective for the treatment of WR following OAGB, with a 97% safety rate and a severe adverse event rate of 3%. Further studies are needed to evaluate the durability of the treatment as well as its potential synergy with newer anti-obesity medications.

## Data Availability

No datasets were generated or analysed during the current study.
